# Global Disparities in Teletherapy Adoption: A Cross-Income Analysis of Mental Health Access

**DOI:** 10.3390/ijerph23020230

**Published:** 2026-02-11

**Authors:** Gloria Nnadwa Alhassan, Arda Ozturkcan, Seyma Caliskan Cavdar

**Affiliations:** 1 Department of Nursing, Faculty of Health Sciences, Istanbul Gelişim University, Istanbul 34310, Türkiye; 2 Department of Nutrition and Dietetics, Faculty of Health Sciences, Istanbul Gelisim University, Istanbul 34310, Türkiye; sozturkcan@gelisim.edu.tr; 3 Department of Economics, Faculty of Economics and Administrative Sciences, Dogus University, Istanbul 34680, Turkey; scaliskan@dogus.edu.tr

**Keywords:** teletherapy, digital mental health, health equity, global disparities, policy analysis, ecological study, healthcare disparities

## Abstract

Mental health disorders affect nearly one billion people worldwide, yet treatment gaps exceed 75% in low- and middle-income countries. Teletherapy has emerged as a scalable solution, but its adoption differs sharply by economic context. This comparative ecological policy analysis used secondary aggregate data from WHO, World Bank, ITU, and national reports to examine teletherapy adoption in low-income (Nigeria, Kenya), middle-income (South Africa, India), and high-income countries (Norway, Canada). Descriptive statistics and simple linear regression were applied, with findings interpreted through the Consolidated Framework for Implementation Research (CFIR), Technology Acceptance Model (TAM), and Diffusion of Innovations theory. High-income countries achieved widespread adoption (>70%), enabled by universal broadband, comprehensive regulation, and strong reimbursement. Middle-income countries showed moderate uptake (15–30%), constrained by rural–urban digital divides and inconsistent policies. Low-income countries reported minimal integration (<5%), limited by unreliable internet, severe workforce shortages, high data costs, and sociocultural barriers. Digital infrastructure, regulatory maturity, and mental health workforce density explained 78% of the cross-country variance in adoption rates (R^2^ = 0.78). Equitable scale-up of teletherapy directly supports SDGs 3, 9, 10, and 17. Targeted investment and cross-income collaboration are essential to prevent digital mental health solutions from exacerbating existing inequities.

## 1. Introduction

Mental health disorders constitute a significant global burden, affecting approximately 970 million people and accounting for 14.6% of total disability-adjusted life years (DALYs) [1]. Despite their high prevalence, access to mental health services remains starkly inequitable, with treatment gaps exceeding 75% in low- and middle-income countries (LMICs) compared to 40% in high-income countries (HICs) [2]. Workforce shortages further exacerbate these disparities, with LMICs averaging fewer than two mental health workers per 100,000 people, compared to over 70 per 100,000 in HICs [3].

Digital health innovations, particularly teletherapy, have emerged as potential solutions to expand mental health access by enabling remote service delivery [4]. The COVID-19 pandemic accelerated the adoption of digital mental health interventions globally, demonstrating their feasibility while exposing critical digital and structural inequities [5]. While HICs rapidly integrated teletherapy into healthcare systems—facilitated by robust infrastructure and policy frameworks—LMICs have faced substantial implementation challenges, including technological deficits, regulatory constraints, and socio-cultural resistance [6].

Although studies have explored the efficacy of teletherapy in high-resource settings, limited research has systematically compared teletherapy adoption across different economic contexts [7]. The absence of such comparative analyses restricts the development of scalable, context-specific strategies for mental health service expansion in LMICs. Furthermore, concerns persist regarding digital health inequities, where socioeconomically disadvantaged populations who bear the highest mental health burden face disproportionate barriers to digital service access [8].

Teletherapy is distinguished from general telehealth by its emphasis on therapeutic applications, emotional presence, and cultural adaptation of psychotherapeutic protocols [9]. These dimensions are uniquely sensitive to digital divides, making income-level comparisons particularly revealing.

Given the plethora of extant literature on the theme under review, this study seeks to address these gaps by conducting a cross-income comparative analysis of teletherapy adoption across low-income (Nigeria, Kenya), middle-income (South Africa, India), and high-income (Norway and Canada) countries. Specifically, it examines the adoption rates, implementation models, and policy frameworks governing teletherapy in different economic settings, secondly the technological, regulatory, and socio-economic factors influencing teletherapy adoption and the context-appropriate strategies to optimize digital mental health service delivery in resource-limited settings. Additionally, by generating evidence-based recommendations, this study seeks to inform equitable digital health policy development, ensuring that teletherapy implementation mitigates rather than exacerbates global mental health disparities.

The reminder of this study proceeds with data and method, and theoretical justification in Section 2. Subsequently, Section 3 presents the empirical results followed by the discussion section in Section 4. Finally, Section 5 renders the concluding remarks and policy implications driven from the study.

## 2. Methods

This study employs a comparative ecological policy analysis using secondary aggregate data to examine teletherapy adoption across low-income (Nigeria, Kenya), middle-income (South Africa, India), and high-income countries (Norway, Canada). Countries were purposively selected based on World Bank income classification, variation in digital infrastructure, and availability of national-level teletherapy indicators and policy documents.

### 2.1. Data Sources and Sources

Publicly available national-level indicators and policy documents (2015–October 2024) were retrieved from WHO Mental Health Atlas, World Bank Development Indicators, ITU World Telecommunication/ICT Indicators, national health ministry reports, and peer-reviewed literature identified through targeted searches in PubMed, Scopus, Web of Science, Google Scholar, and WHO IRIS using terms combining “teletherapy”, “telemental health”, country names, and “adoption/policy/implementation”. Only sources providing quantitative estimates of adoption rates, workforce density, broadband penetration, or regulatory status for the six selected countries were used. No formal risk-of-bias assessment was applied, as the analysis relies on official aggregate statistics rather than individual studies. Further insights are rendered in Table 1.

### 2.2. Analytical Approach

Data were organised in a structured matrix by country and income group. Key indicators were tabulated for descriptive comparison. Simple linear regression was performed on the seven country-level observations (six countries + pooled HIC average) to identify predictors of adoption.

### 2.3. Theoretical Frameworks

The study is grounded in three key theoretical models:

- Consolidated Framework for Implementation Research (CFIR)—domains used: Outer Setting (national policies, financing), Inner Setting (organisational culture, workforce readiness), Innovation Characteristics (relative advantage, compatibility, cost) [10].
- Rogers’ Diffusion of Innovation Theory: This theory explains how new ideas and technologies spread within a society. Rogers [11] posits that adoption occurs in stages: knowledge, persuasion, decision, implementation, and confirmation. Key factors influencing adoption include relative advantage, compatibility, complexity, trialability, and observability [11]. Given the disparities in teletherapy uptake between LICs, MICs, and HICs, this framework helps elucidate why certain regions experience faster diffusion of digital mental health services than others.
- Technology Acceptance Model (TAM): TAM, developed by Davis, ref. [12] explains how users come to accept and use a technology. It highlights two primary determinants: perceived usefulness (PU) and perceived ease of use (PEOU). This model is particularly relevant in evaluating the willingness of healthcare providers and patients to adopt teletherapy services, especially in settings with limited digital literacy or infrastructural challenges. The integration of CFIR, TAM, and Diffusion of Innovation theory has been made explicit, detailing which constructs guided coding, comparison, and interpretation.

The integration of CFIR, TAM, and Diffusion of Innovation theory has been made explicit, detailing which constructs guided comparison and interpretation. By integrating these theoretical perspectives, the study provides a multi-faceted analysis of teletherapy adoption across individual, organisational, and systemic levels [9,10,13,14].

## 3. Results

Substantial disparities exist in teletherapy adoption across economic settings. High-income countries (HICs), such as Norway, report integration rates exceeding 70%, while Canada similarly reports 68% adoption in 2023–2024 [15] supported by strong digital health policies, high provider capacity, and strong public trust in telehealth services [14,15]. In contrast, middle-income countries (MICs), including South Africa and India, exhibit moderate adoption (15–30%) due to emerging digital strategies and urban–rural disparities in accessibility [16]. Low-income countries (LICs), such as Nigeria and Kenya, report adoption rates below 5%, constrained by inadequate infrastructure, policy fragmentation, and workforce shortages [16,17].

The feasibility of teletherapy implementation is shaped by significant disparities in digital infrastructure. HICs benefit from universal broadband coverage and government-backed digital health initiatives, ensuring widespread service availability [5]. MICs show moderate connectivity, with rural–urban gaps in access. LICs face severe constraints, including unreliable networks, high data costs, and limited digital literacy, restricting teletherapy accessibility to small-scale initiatives.

HICs exhibit strong provider networks, enabling seamless teletherapy integration. MICs demonstrate variable adoption, with urban areas leveraging teletherapy while rural regions remain underserved. LICs experience critical shortages in trained mental health professionals, exacerbating access disparities [3,18].

The charts and table below summarize the disparities in teletherapy adoption across economic settings, highlighting variations in implementation levels, infrastructure barriers, and regulatory frameworks.

**Figure 1:**
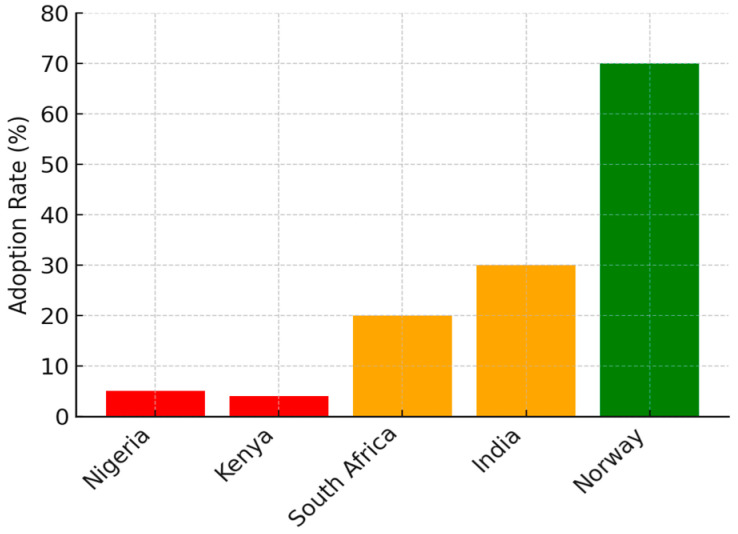
Teletherapy adoption rates.

Figure 1 shows the teletherapy adoption rates by economic context—adoption is lowest in LICs—Nigeria and Kenya, (~5%), moderate in MICs—South Africa and India (~30%), and highest in HICs—Norway (~70%).

**Figure 2:**
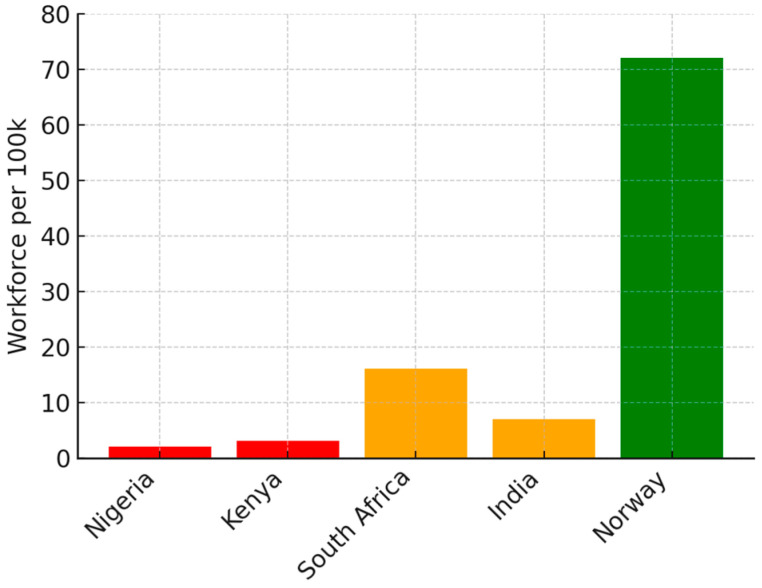
Mental health workforce per 100,000 people.

Figure 2 shows the mental health workforce per 100,000 people—LICs—Nigeria and Kenya, have a severe shortage (~2.5 per 100 k), MICs—South Africa and India, have moderate numbers (~15 per 100 k), while HICs—Norway, have robust staffing (~72 per 100 k).

Table 2 presents further disparities, showing that high-income countries (HICs) have advanced teletherapy strategies, MICs demonstrate emerging but inconsistent adoption, and LICs struggle with infrastructure challenges.

Simple linear regression on aggregate country-level data showed that log GDP per capita, broadband penetration, and mental health workforce density together explained 78% of the variance in reported teletherapy adoption rates (R^2^ = 0.78, *p* < 0.01; Table 3).

Results from this study show that technology, policy and regulation and equity considerations play a major role in facilitating or limiting teletherapy adoption.

- Technological Barriers: Limited broadband coverage, high device costs, and low digital literacy hinder adoption, particularly in LICs and rural MICs. The lack of culturally and linguistically appropriate teletherapy platforms further reduces engagement, disproportionately affecting marginalized populations [8].
- Policy and Regulatory Challenges: Fragmented or absent regulatory frameworks in LICs limit teletherapy scale-up, while MICs show emerging but inconsistent policies. HICs have well-established digital health laws, facilitating reimbursement mechanisms and data privacy protections that support teletherapy expansion [19].
- Equity Considerations: The risk of digital exclusion remains a significant concern. Without targeted interventions, existing socioeconomic and geographic disparities may be exacerbated, reinforcing health inequities rather than addressing them [8,20].

## 4. Discussion and Policy Implications

This comprehensive analysis of teletherapy implementation across diverse economic contexts reveals substantial and multifaceted disparities influenced by structural, policy, and sociocultural factors. While high-income countries (HICs) like Norway demonstrate sophisticated teletherapy integration with adoption rates exceeding 70%, and Canada reports 68% teletherapy adoption in 2023–2024, reinforcing HIC patterns, middle-income countries (MICs) such as India and South Africa show moderate but inconsistent integration (15–30%), and low-income countries (LICs) including Nigeria and Kenya remain at marginal implementation levels (<5%) [15,20,21]. 

These findings extend beyond previous observations of mental health service gaps, ref. [2] uncovering a complex interplay of “digital determinants of health” that fundamentally shape teletherapy accessibility and effectiveness [5,22]. The stark variation in internet infrastructure—nearly universal in HICs versus limited, costly, and unreliable in LICs—directly impacts service delivery capacity, aligning with Crawford and Serhal’s [5,23,24] Digital Health Equity Framework while providing empirical evidence of its manifestation across economic strata. The analysis reveals that these digital determinants operate not merely as technological barriers but as structural inequities embedded within broader socioeconomic systems.

Unlike previous digital health research focused primarily on technological adoption factors [16], this study demonstrates that teletherapy implementation follows complex, context-dependent trajectories rather than the linear diffusion patterns predicted by traditional innovation diffusion theories. Implementation success appears contingent on the convergence of multiple factors: digital infrastructure readiness, healthcare workforce capacity, regulatory frameworks, financial mechanisms, and cultural acceptance—factors that vary significantly across and within economic contexts.

National policy environments critically influence implementation success, with considerable implications for teletherapy integration. Countries with comprehensive digital health strategies and well-articulated teletherapy-specific regulations (predominantly HICs) demonstrate more effective, equitable, and sustainable integration compared to those with fragmented or absent policy frameworks (primarily LICs), consistent with Walt and Gilson’s [19,25,26] policy analysis paradigm. This regulatory disparity manifests tangibly in healthcare workforce capacity, with marked variations in mental health professionals per 100,000 populations (Norway: 72; South Africa: 16; India: 7; Nigeria: 2; Kenya: 3), exceeding differences documented in the WHO Mental Health Atlas [3,15,26,27,28] and suggesting that digital solutions alone cannot overcome fundamental workforce limitations.

Sociocultural factors—including mental health stigma, trust in digital platforms, and preferences for traditional healing approaches—play equally determinative roles in shaping adoption patterns. Unlike assumptions that technology automatically increases service utilization [29,30] the findings suggest that cultural context profoundly mediates teletherapy acceptance and engagement. In settings where mental illness remains highly stigmatized or associated with traditional and supernatural beliefs, digital interventions face resistance beyond technological access barriers, necessitating culturally adapted implementation approaches rarely featured in current teletherapy platforms.

The pronounced urban-rural divide in teletherapy access, particularly evident in MICs, reinforces existing geographical inequities in mental healthcare provision, contradicting technological determinism narratives that suggest digital solutions inherently reduce disparities [5,6,24]. The data indicates that without targeted interventions addressing both technical infrastructure and socioeconomic barriers, teletherapy may paradoxically exacerbate rather than mitigate mental health inequalities a concerning possibility not adequately addressed in previous implementation research [21]. This challenge is particularly salient for marginalized populations who face intersecting barriers of limited connectivity, affordability constraints, and lower digital literacy.

Based on these multidimensional findings, an equity-centered implementation framework emerges, comprising three essential and interdependent policy framework (1) Infrastructure: National broadband funds (e.g., India’s BharatNet model) + zero-rated mental health data [31,32]; (2) Regulation: LIC telemedicine licensure reciprocity with HIC boards (e.g., Norway–Kenya twinning) [15,23]; (3) Workforce: Task-shifting to community health workers with 6-week digital CBT training (WHO Mental Health Gap Action Programme (mhGAP)) [33]. This framework acknowledges that successful teletherapy deployment requires simultaneous attention to both technological and human dimensions within specific socioeconomic contexts, moving beyond technocentric approaches that have characterized much digital health implementation to date.

Addressing these challenges aligns with several Sustainable Development Goals (SDGs), including SDG 3 (Good Health and Well-being) by promoting equitable mental health access, SDG 9 (Industry, Innovation, and Infrastructure) through digital health investment, and SDG 10 (Reduced Inequalities) by bridging healthcare access gaps. Additionally, SDG 4 (Quality Education) is relevant, as digital literacy and professional training are critical for scaling teletherapy, while SDG 17 (Partnerships for the Goals) highlights the need for global collaboration in developing sustainable and culturally adapted teletherapy models. Strengthening global collaboration and leveraging lessons from successful implementations can facilitate more inclusive and sustainable digital mental health strategies worldwide.

Several limitations of this study warrant acknowledgment. Despite examining multiple countries across income groups, data availability constrains generalizability to additional regions and healthcare contexts. Additionally, the rapid evolution of digital health technologies means these findings represent a contextual snapshot rather than definitive long-term trends. Also the review included only English-language sources and aggregated national data, masking subnational variation. Causal claims are limited by ecological design. The cross-sectional nature of the analysis also limits causal inferences regarding specific implementation factors and outcomes. Future research should explore artificial intelligence applications in expanding teletherapy accessibility, particularly in resource-constrained settings; examine lived experiences of underrepresented populations using mixed-methods approaches; and investigate sustainable financing models for equitable implementation beyond donor-dependent initiatives.

## 5. Conclusions

This study reveals profound global disparities in teletherapy adoption that are driven by digital infrastructure, regulatory maturity, and mental health workforce capacity rather than clinical need. High-income countries have successfully integrated teletherapy into routine care, while low- and middle-income countries remain largely excluded despite bearing the greatest burden of mental disorders. Without deliberate, equity-centered policies—including targeted infrastructure investment, regulatory transformation, and task-shifting models—teletherapy risks widening rather than narrowing the global mental health treatment gap. Strengthening cross-income collaboration and aligning digital mental health strategies with SDGs 3, 9, 10, and 17 offer a viable path toward more inclusive and sustainable service delivery worldwide.

## Figures and Tables

**Table 1:** Search strategy & inclusion criteria.

Component	Description
Databases searched	PubMed, Scopus, Web of Science, Google Scholar, WHO IRIS
Additional sources	National health ministries, World Bank, ITU, WHO country reports, hand-searching of references
Publication dates	January 2015–October 2024
Language	English only
Inclusion criteria	Reports teletherapy/telemental health adoption, implementation, policy, or barriers Focus on Nigeria, Kenya, South Africa, India, Norway, or Canada
Exclusion criteria	Pure efficacy trials without implementation data Asynchronous text-only interventions Non-English, pre-2015, conference abstracts only
Total sources in synthesis	69 (42 peer-reviewed/grey literature + 27 policy documents)

Source: authors’ compilation.

**Table 2:** Comparative analysis of teletherapy adoption and infrastructure across economic contexts.

Category	Low-Income Countries (LICs—Nigeria, Kenya)	Middle-Income Countries (MICs—South Africa, India)	High-Income Country (HIC—Norway)
Implementation Level	Fragmented, pilot-level, donor-funded	Structured, emerging digital strategies	Comprehensive, national strategy
Internet Connectivity	Limited, digital divide	Moderate, urban-rural disparities	Universal access
Infrastructure Barriers	Inadequate broadband, high costs, unreliable power	Moderate broadband, urban-rural disparities	Advanced infrastructure
Technology Accessibility	Low smartphone penetration, high device costs	Moderate smartphone penetration	High accessibility
Regulatory Framework	Limited policies, fragmented strategies	Emerging telemedicine guidelines	Comprehensive digital health laws
Reimbursement & Financing	Minimal insurance support	Varies, emerging mechanisms	Strong financial support
Cultural Acceptance	Low trust, strong influence of traditional beliefs	Moderate, evolving perceptions	High trust in digital health
Mental Health Awareness	Low, stigma prevalent	Improving awareness, stigma remains	High literacy, well-integrated
Equity Implications	High risk of exclusion, digital divide	Potential for inclusion with challenges	Mostly inclusive, some gaps remain

Source: authors’ compilation.

**Table 3:** Predictors of national teletherapy adoption rate (simple linear regression on aggregate country-level data).

Predictor	β	SE	*p*-Value	Model R^2^
GDP per capita (log)	0.62	0.09	<0.001	
Broadband penetration (%)	0.41	0.11	0.002	
Mental health workers/100 k	0.33	0.08	0.004	0.78

Sources: Adoption rates (national health surveys & reports 2021–2024); GDP (World Bank); broadband (ITU World Telecommunication Indicators 2023); workforce (WHO Mental Health Atlas 2020–2023). N = 7 country aggregates (Nigeria, Kenya, South Africa, India, Norway, Canada, plus pooled HIC average for robustness) [1,15,19].

## Data Availability

The data supporting the findings of this manuscript are available from the corresponding authors upon reasonable request.
